# p53 functional states are associated with distinct aldehyde dehydrogenase transcriptomic signatures

**DOI:** 10.1038/s41598-020-57758-5

**Published:** 2020-01-23

**Authors:** Shanying Gui, Xiujie Xie, Wendi Q. O’Neill, Kate Chatfield-Reed, Jun-Ge Yu, Theodoros N. Teknos, Quintin Pan

**Affiliations:** 1 University Hospitals Seidman Cancer Center, Cleveland, OH 44106 USA; 2 Department of Otolaryngology-Head and Neck Surgery, Case Western Reserve University, School of Medicine, Cleveland, OH 44106 USA; 3 Department of Internal Medicine, The Ohio State University Wexner Medical Center, Columbus, OH USA; 4 Department of Otolaryngology-Head and Neck Surgery, The Ohio State University Wexner Medical Center, Columbus, OH USA; 5 Case Comprehensive Cancer Center, Case Western Reserve University, School of Medicine, Cleveland, OH 44106 USA

**Keywords:** Cancer stem cells, Head and neck cancer

## Abstract

p53 and aldehyde dehydrogenase (ALDH) have been implicated in key tumorigenesis processes including cancer initiating cell (CIC) maintenance; however, the relationship between these two mediators remains poorly defined. In this study, ALDH isoform expression diversity was revealed in CICs with disparate p53 functional states: gain of function, high risk p53 mutation (p53HRmut) and wildtype p53 (p53WT) inactivated by the human papillomavirus 16 (HPV16) E6 oncogene. Interrogation of head and neck squamous cell carcinoma (HNSCC) cell lines and patient tumors showed that HPV16+/p53WT cases have higher ALDH variance score (AVS), a measure of tumor ALDH isoform expression diversity, compared to HPV−/p53HRmut cases (p = 0.03). AVS and several individual ALDH isoforms were associated with prognosis in HPV16+/p53WT HNSCC but not in HPV−/p53HRmut HNSCC. Knockdown of the dominant ALDH isoform in high AVS HNSCC depleted the CIC pool *in vitro* and *in vivo*. Our results demonstrate that p53 functional states are associated with distinct ALDH isoform transcriptomic signatures. Moreover, tumor ALDH profiling may provide insight on which ALDH isoform to target in high AVS HNSCC tumors to deplete the CIC population.

## Introduction

A frequent genetic event in the tumorigenesis cascade is aberrant p53 function, either loss of wildtype p53 (p53WT) function or gain of function, high-risk p53 mutation (p53HRmut). p53WT controls normal stem cell homeostasis and evidence exists to support p53WT dysfunction in cancer initiating cell (CIC) expansion and maintenance^1^. Nanog, a core embryonic stem cell transcription factor, known to modulate normal stem cell and CIC pluripotency is negatively regulated by p53WT^2–4^. In addition, CD44 and CD133, two well-recognized markers for CICs, are repressed by p53WT through direct promoter occupancy^5,6^. A logical extension of these studies is that loss of p53WT function will likely result in CIC expansion through multiple mechanisms to drive cancer promotion and progression. In contrast to p53WT, literature on p53HRmut in this space is scant. A recent paper reported that p53HRmut is more efficient than p53WT deficiency to facilitate somatic cell reprogramming; however, these p53HRmut reprogrammed cells exhibit genetic instability and have a higher potential for malignant transformation with tumor initiating properties^7^.

Aldehyde dehydrogenase (ALDH) is a superfamily consisting of 19 evolutionarily conserved isoforms and its activity is used as a functional assay to identify normal stem cells and CICs. Based on initial work, it was generally believed that ALDH1A1 is indispensable for high ALDH activity to maintain the CIC pool^8–10^. However, depending on the cell line or anatomical site, there is now compelling evidence that other ALDH isoforms may contribute to or be primarily responsible for elevated ALDH activity in CICs^11–15^. A relationship between p53 and ALDH was revealed when p53HRmut was shown to act as a transcription factor to drive ALDH1A1 transcription to confer a novel gain of function effect on CIC maintenance and/or expansion^16^. However, the influence of p53 functional states on the ALDH gene family in the context CICs and cancer, in general, remains poorly understood.

Head and neck squamous cell carcinomas (HNSCCs) are cancers from various anatomical site in the head and neck region and can be subdivided based on two major etiologic factors: smoking/alcohol use and high-risk human papillomavirus (HPV), in particular HPV16. HPV− HNSCCs are driven by smoking and alcohol use, and often harbor p53 genomic alterations, mostly gain of function, missense mutations. In contrast, HPV + HNSCCs have p53WT that is inactivated by the HPV oncogene, E6. These two distinct etiologies offer an unique opportunity to explore the relationship between p53 and ALDH without the use of genetic engineering approaches. Using HPV− and HPV16+ cell lines and patient tumors, we showed that p53 functional states have differential ALDH isoform expression diversity at the CIC and bulk tumor levels. HPV16+/p53WT tumors tend to display a dominant ALDH isoform expression pattern with enrichment of a particular ALDH family member. Interestingly, targeting the dominant ALDH isoform in high AVS HNSCC cell lines resulted in robust depletion of the CIC population. Our work demonstrates that p53 functional states are associated with distinct ALDH isoform transcriptomic signatures and suggests that tumor ALDH profiling may identify the isoform responsible for CIC maintenance in high AVS HNSCC.

## Results

### ALDH isoform transcriptomic profiles in CICs from HPV16+ and HPV− HNSCC cell lines

**Figure 1:**
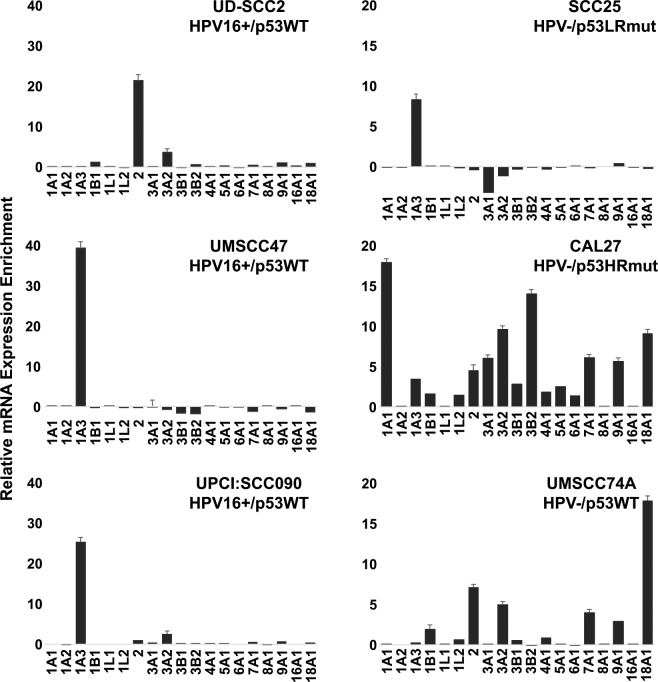
ALDH isoform transcriptomic profiles in CICs from HPV16+ and HPV− HNSCC cell lines. A panel of HPV16+ and HPV− HNSCC cell lines with distinct p53 functional states were sorted for ALDH^high^ and ALDH^low^ subsets, and assessed for expression of individual ALDH isoforms. Data is presented as relative mRNA expression enrichment in the ALDH^high^ CIC population (mean ± s.e.m.; n = 3).

The HPV16+ HNSCC cell lines, UD-SCC2, UMSCC47, and UPCI-SCC090, have wildtype p53 that is inactivated by HPV16E6^17^. The HPV− HSNCC cell lines have distinct p53 states; CAL27 has gain of function, high risk mutant p53 (H193L), SCC25 has loss of function p53 due to the deletion of two base pairs in codon 209, and UMSCC74A has wildtype p53^18^. ALDH activity measured using the ALDEFLUOR assay is a well-established methodology to identify and quantitate CICs in hematologic and solid malignancies. ALDH^high^ CIC fraction varied across our panel of HNSCC cell lines (Supplemental Fig. 1). We collected ALDH^high^ (top 5%) and ALDH^low^ (bottom 5%) populations to determine the ALDH isoform expression signature in CICs from these cell lines. As shown in Fig. 1a, ALDH^high^ CICs from HPV16+/p53WT HNSCC displayed a dominant ALDH isoform expression signature; ALDH1A3 was enriched in UMSCC47 and UPCI-SCC090 CICs, whereas, ALDH2 was the main isoform enriched in UD-SCC2 CICs. Similarly, in SCC25, a HPV− HNSCC cell line with truncated, loss of function p53, ALDH^high^ CICs were enriched for a single ALDH isoform, ALDH1A3. In contrast, high ALDH isoform expression diversity was shown in ALDH^high^ CICs from HPV−/p53HRmut CAL27. HPV−/p53WT UMSCC74A CICs represent an intermediate group and had moderate ALDH isoform expression diversity.

### HPV16+/p53WT HNSCC have higher ALDH variance score than HPV−/p53HRmut HNSCC

**Figure 2:**
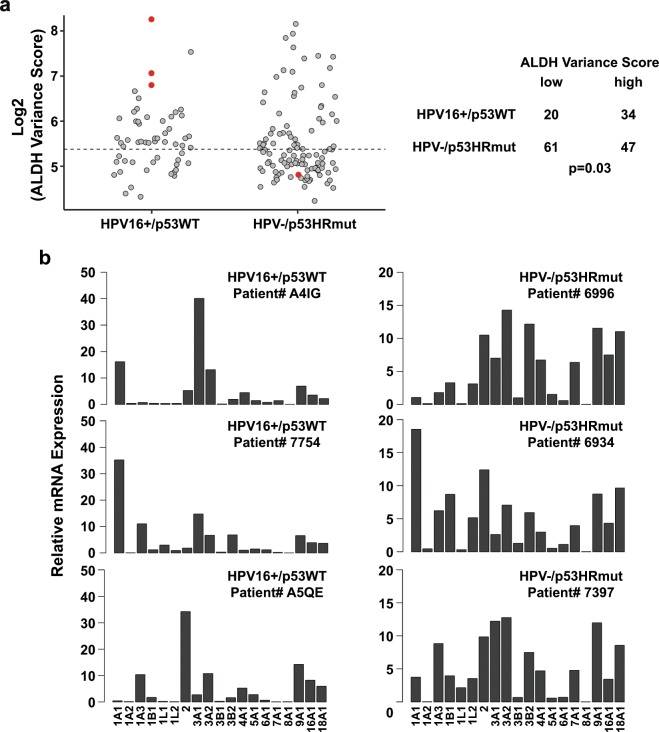
HPV16+/p53WT HNSCCs have higher ALDH variance score than HPV−/p53HRmut HNSCCs. (**a**) ALDH variance score (AVS). AVS was calculated for HPV16+/p53WT and HPV−/p53HRmut HNSCC cell lines and primary tumors. Cell lines are represented by red circles and primary tumors from the TCGA HNSCC cohort are represented by grey circles. The dashed line represents the median AVS for the combined cohort. Median AVS for the combined cohort was used to stratify into two groups: low AVS and high AVS (p = 0.03, Fisher’s exact test). (**b**) ALDH isoform expression profiles from representative HPV16+/p53WT and HPV−/p53HRmut HNSCC patients.

Our results using HNSCC cell lines suggest that HPV status and p53 functional states may drive distinct ALDH expression signatures. In order to extend these findings from cell lines to patient tumors, we developed a mathematical algorithm, ALDH variance score (AVS), as an approach to quantitate the ALDH isoform expression variance in individual samples. HPV16+/p53WT HNSCC cell lines had AVS >100; 109.6 for UD-SCC2, 313.1 for UMSCC47, and 137.4 for UPCI-SCC090. HPV− SCC25 expresses truncated, loss of function p53 and thus, has an inactivated p53 functional state analogous to HPV16-driven inactivation of p53WT. Similar to HPV16+/p53WT cell lines, SCC25 had a high AVS of 175.7. In comparison, HPV−/p53HRmut CAL27 HNSCC cell line had a low AVS of 28.4. Next, the AVS algorithm was applied to the HPV16+ and HPV− cohorts from the TCGA HNSCC dataset. The difference in AVS was unremarkable between HPV16+ and HPV− tumors (Supplemental Fig. 2a). Moreover, when tumors were binned based on p53 mutational status, AVS distribution was comparable across the p53WT, p53LRmut, and p53HRmut cohorts (Supplemental Fig. 2b). We focused our work on HPV16+/p53WT and HPV−/p53HRmut since these two distinct entities are frequently presented in the clinic. As shown in Fig. 2a, HPV16+/p53WT tumors are associated with high AVS, and HPV−/p53HRmut tumors tend to have low AVS (p = 0.03, Fisher’s exact test). Representative HPV16+/p53WT HNSCC tumors display a dominant ALDH isoform expression pattern with high AVS, whereas, representative HPV−/p53HRmut HNSCC tumors show greater ALDH isoform expression diversity with low AVS (Fig. 2b).

### ALDH variance score is associated with stemness index

**Figure 3:**
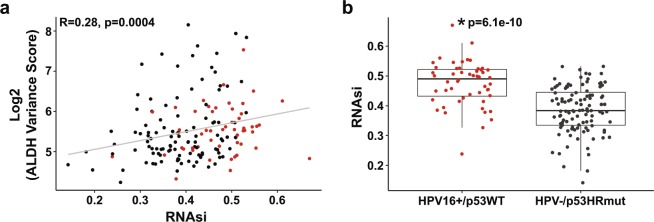
ALDH variance score is associated with stemness index. (**a**) AVS and mRNAsi. High AVS is correlated with a high mRNAsi (r = 0.28, p = 0.0004, Spearman correlation). (**b**) mRNAsi in HPV16+/p53WT and HPV−/p53HRmut tumors. Data are presented as box plots (p = 6.1 × 10^−10^, Wilcoxon test).

Direct quantitation of the CIC pool in individual TCGA HNSCC tumors is not feasible so we used the PanCancer Atlas stemness index (mRNAsi)^19^ as a tool to infer relative CIC frequency. High AVS is associated with a high mRNAsi (r = 0.28, p = 0.0004) in this TCGA HNSCC cohort (Fig. 3a). Moreover, mean mRNAsi was higher in HPV16+/p53WT tumors than in HPV−/p53HRmut HNSCC tumors (p = 6.1 × 10^−10^). This is consistent with a previous report showing that HPV16+/p53WT HNSCCs have a higher intrinsic CIC frequency than HPV−/p53HRmut HNSCC^20^ and thus, supports the potential utility of mRNAsi to infer relative CIC frequency from global transcriptome in patient tumors.

### ALDH variance score is associated with survival in HPV16+/p53WT HNSCC

**Figure 4:**
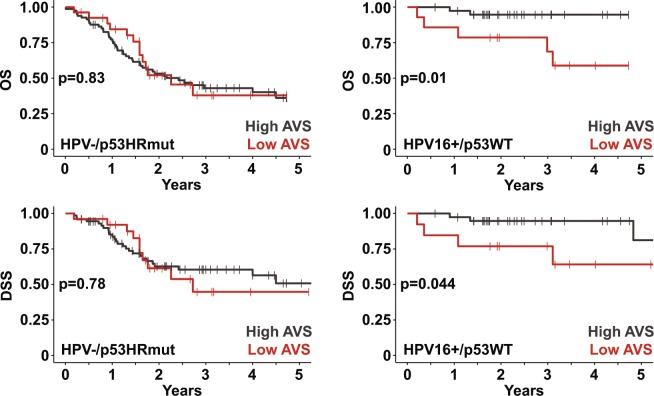
ALDH variance score is a prognostic biomarker in HPV16+/p53WT HNSCC. Five-year Kaplan-Meier plots for overall and disease-specific survival in HPV16+/p53WT and HPV−/p53HRmut cohorts. Cohorts were stratified based on AVS: high AVS denotes top 75% and low AVS denotes bottom 25%. Log-rank test was used to compare the Kaplan-Meier plots.

Since HPV16+/p53WT patients have superior overall survival (OS) and disease-specific survival (DSS) compared to HPV−/p53HRmut patients (Supplemental Fig. 3), the influence of AVS on prognosis was analyzed for each cohort separately. In Fig. 4, in the HPV16+/p53WT setting, high AVS patients had superior OS (log-rank, p = 0.01) and DSS (log-rank, p = 0.044) compared to low AVS patients. However, the prognostic utility of AVS was not observed in the HPV−/p53HRmut cohort. Next, we took a step further and investigated the clinical impact of individual ALDH isoforms. High ALDH1A3, ALDH3B1, ALDH7A1, and ALDH18A1 correlated with poor prognosis, whereas, elevated ALDH2, an isoform with tumor suppressive actions, was associated with better OS in HPV16+/p53WT patients (Table 1). None of the ALDH isoforms was prognostic in the HPV−/p53HRmut setting.

**Table 1:** Select ALDH isoforms are prognostic biomarkers in HPV16+/p53WT HNSCC.

HPV16+/p53WT			HPV−/p53HRmut	
	HR (95% CI)	p-value	HR (95% CI)	p-value
ALDH1A1	0.94 (0.82, 1.08)	0.376	0.99 (0.96, 1.02)	0.643
ALDH1A2	1.02 (0.81, 1.27)	0.886	0.74 (0.22, 2.50)	0.630
ALDH1A3	1.17 (1.01, 1.36)	0.033	1.01 (0.98, 1.05)	0.438
ALDH1B1	1.13 (0.91, 1.40)	0.269	0.99 (0.91, 1.08)	0.889
ALDH1L1	1.07 (0.81, 1.42)	0.617	1.38 (0.81, 2.36)	0.234
ALDH1L2	1.16 (0.35, 3.85)	0.813	0.94 (0.79, 1.11)	0.450
ALDH2	0.85 (0.74, 0.98)	0.023	0.99 (0.97, 1.03)	0.937
ALDH3A1	1.02 (0.97, 1.08)	0.425	1.00 (0.99, 1.02)	0.781
ALDH3A2	0.85 (0.71, 1.00)	0.056	0.97 (0.91, 1.04)	0.381
ALDH3B1	3.15 (1.25, 7.96)	0.015	1.08 (0.75, 1.57)	0.665
ALDH3B2	1.07 (0.97, 1.18)	0.205	0.99 (0.97, 1.02)	0.712
ALDH4A1	1.08 (0.88, 1.33)	0.449	0.99 (0.92, 1.06)	0.715
ALDH5A1	0.97 (0.60, 1.59)	0.915	1.15 (0.90, 1.47)	0.267
ALDH6A1	1.16 (0.25, 5.49)	0.851	0.95 (0.53, 1.70)	0.857
ALDH7A1	1.25 (1.03, 1.50)	0.021	1.02 (0.96, 1.09)	0.576
ALDH8A1	1.10 (0.80, 1.30)	0.180	0.52 (0.09, 2.82)	0.445
ALDH9A1	0.90 (0.71, 1.14)	0.390	1.04 (0.96, 1.12)	0.351
ALDH16A1	0.91 (0.64, 1.31)	0.624	0.98 (0.90, 1.07)	0.704
ALDH18A1	1.33 (1.07, 1.65)	0.011	0.99 (0.93, 1.06)	0.889

### Targeting the dominant ALDH isoform in high AVS HNSCC depletes the CIC pool

**Figure 5:**
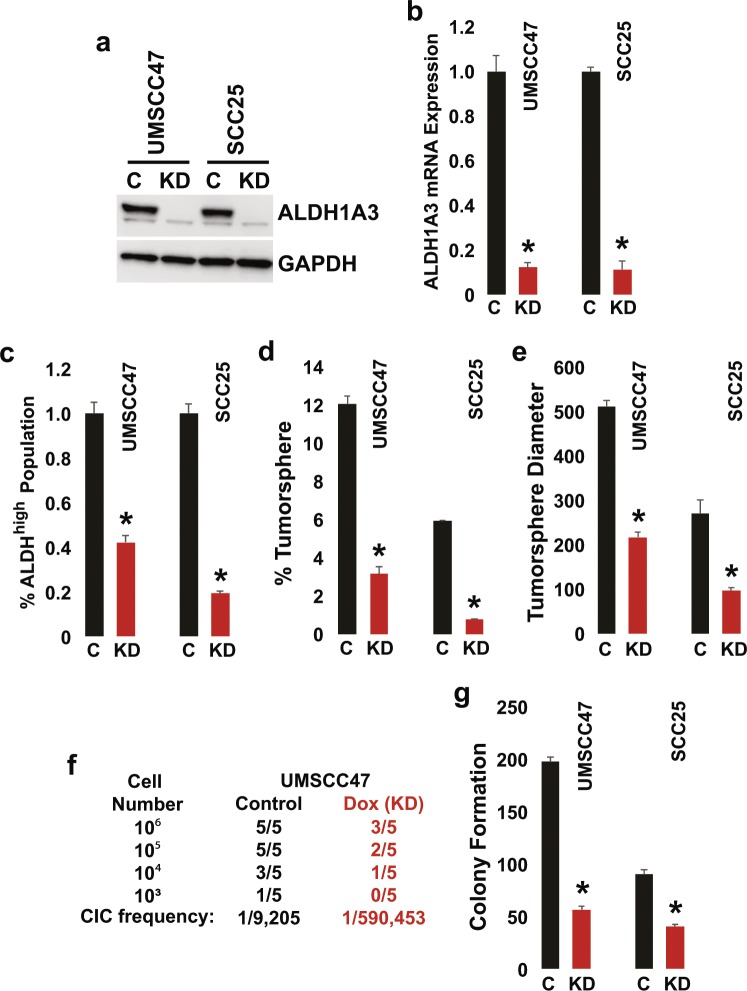
Targeting the dominant ALDH isoform in high AVS HNSCC depletes the CIC pool. UMSCC47 and SCC25 cells were transduced with the inducible pLV-RNAi/shRNA-ALDH1A3 and polyclonal cell populations were collected. Cells were stimulated with doxycycline at 1000 ng/ml for all the experiments. (**a**) ALDH1A3 protein levels. Cell lysates were immunoblotted with anti-ALDH1A3 and GAPDH antibodies. Representative image is cropped. (**b**) ALDH1A3 mRNA expression. ALDH1A3 and GAPDH expression was determined using qPCR with TaqMan primers. Data were normalized to GAPDH and are presented as mean ± s.e.m. (n = 3, *p < 0.05, two-tailed Student’s t-test). (**c**) ALDH^high^ CIC population. Cells were analyzed by FACS and ALDH^high^ CIC population was quantitated using the ALDEFLUOR assay. Data are presented as mean ± s.e.m. (n = 3, *p < 0.05, two-tailed Student’s t-test). (**d,e**) Tumorsphere formation efficiency and diameter. Cells were harvested, seeded on low-attachment plates in a defined, serum-free culture medium, and tumorspheres were allowed to grow. Tumorsphere formation efficiency was calculated as the number of tumorspheres formed divided by the original number of cells seeded. Data are presented as mean ± s.e.m. (n = 3, *p < 0.05, two-tailed Student’s t-test). (**f**) *In vivo* cancer initiating cell **f**requency. Indicated number of cells were implanted subcutaneously in the flanks of NSG mice. Tumor incidence (palpable tumor of any size) was noted over the course of the experiment. Cancer initiating cell (CIC) frequency was calculated using the L-Calc program. (**g**) Clonogenic survival. Cells were plated and allowed to grow in complete media for 10 days. Subsequently, colonies were fixed, stained with crystal violet, and counted. Data are presented as mean ± s.e.m. (n = 3, *p < 0.05, two-tailed Student’s t-test).

We hypothesized that high AVS is indicative of a homogenous CIC pool and thus, targeting the dominant ALDH isoform may result in contraction of the CIC population. Tetracycline-inducible ALDH1A3 shRNA polyclonal UMSCC47 and SCC25 cells were generated to determine if the dominant ALDH isoform is indispensable for CIC maintenance in representative HPV16+ and HPV− HNSCC with high AVS (Fig. 5). ALDH1A3 mRNA expression and protein levels were substantially reduced following induction with doxycycline. Knockdown of ALDH1A3 depleted the ALDH^high^ CIC population by 57% in UMSCC47 and 79% in SCC25. Furthermore, tumorsphere formation efficiency and tumorsphere diameter were reduced; 71% and 58% inhibition in UMSCC47 and, 83% and 67% inhibition in SCC25, respectively. *In vivo* limiting dilution assay in NSG mice showed that ALDH1A3 knockdown dramatically depleted the CIC population by > 60-fold in UMSCC47. CIC frequency was reduced from 1/9,205 to 1/590,453 (p < 0.001).

## Discussion

There are hints in the literature that p53 functional states regulate ALDH to modulate the CIC pool. Reactivation of p53WT in HPV16+/p53WT HNSCC depleted the ALDH^high^ CIC pool^20^. Knockout of p53HRmut in SW480 colorectal carcinoma cells resulted in CIC population contraction and reduction of ALDH1A1 expression^16^. Moreover, p53^−/−^ RKO cells showed higher levels of ALDH1A3 compared to its isogenic p53^+/+^ counterpart^16^. These findings indicate that perturbations of p53 functional states have a consequence on CIC maintenance and regulation of certain ALDH isoforms. However, since these studies assessed only a select number of ALDH isoforms, the connection between p53 and ALDH in cancer remains poorly defined.

In this study, we assessed the expression profile of the entire ALDH gene family in HNSCC cell lines and primary tumors with defined HPV and p53 statuses. A dominant ALDH isoform expression signature was shown in HPV16+/p53WT CICs. In contrast, HPV−/p53HRmut CAL27 had CICs with considerable ALDH isoform expression diversity; seven isoforms were enriched by >5-fold. Using AVS as a measure of ALDH isoform expression diversity, analysis of the TCGA HNSCC dataset indicated that HPV16+/p53WT tumors have higher AVS compared to HPV−/p53HRmut tumors revealing that the differences in ALDH expression signature between p53 functional states may not be limited to the CIC subset but extend to the bulk tumor cell population as well. These findings led to the speculation that CIC frequency and/or genomic homogeneity is appreciably higher in HPV16+/p53WT tumors than in HPV−/p53HRmut tumors and thus, transcriptomes of HPV16+/p53WT tumors may better reflect the CIC population. This concept is supported by several pieces of evidence: (a) HPV16 preferentially infects basal cells in the squamous epithelium and these undifferentiated, isogenic cells are likely to be the cell of origin for HPV16+/p53WT tumors, (b) HPV16+/p53WT tumors have higher CIC frequency^20^ and mRNAsi (Fig. 3) than HPV−/p53HRmut tumors, and (c) HPV16+/p53WT tumors have lower aneuploidy score^21^ and mutant allele tumor heterogeneity (MATH)^22^ than HPV−/p53HRmut tumors (Supplemental Fig. 4).

The ALDH superfamily consists of 19 evolutionarily conserved isoforms recognized to oxidize aldehydes to carboxylic acids^23^. In addition to aldehyde metabolism, ALDHs are involved in a plethora of cellular processes which influence tumorigenesis, including retinoic acid (RA) synthesis and signaling, ultraviolet light absorption, hydroxyl radical scavenging, and antioxidant activity^24,25^. Multiple groups have investigated and shown select ALDH isoforms, in particular ALDH1 members, as prognostic biomarkers in a spectrum of solid malignancies^26–28^. We assessed the entire ALDH family and found a select number of isoforms, ALDH1A3, ALDH2, ALDH3B1, ALDH7A1, and ALDH18A1, to be associated with survival in HPV16+/p53WT HNSCC. ALDH2 has tumor suppressive actions and polymorphisms in this gene is associated with increased risk to a number of alcohol-related cancers^29^. The pro-tumorigenic role of ALDH1A3 in cancer is well-described and its utility as a prognostic biomarker is beginning to emerge^11,14,30^. Scant literature exists to link ALDH3B1, ALDH7A1, and ALDH18A1 to cancer, although, these studies provide initial evidence that these isoforms favor tumorigenesis^31–33^. Additional work is needed to improve our understanding of these poorly studied ALDH isoforms in tumorigenesis, stemness, and treatment relapse in HPV16-driven malignancies.

AVS was developed as a quantitative tool to capture ALDH isoform expression diversity from bulk tumor transcriptomic datasets. An initial concern was that AVS may just be capturing global genomic and transcriptomic differences. However, this potential issue was mitigated based on our analyses showing that AVS was not correlated with aneuploidy score or MATH (Supplemental Fig. 4) and moreover, assessment of the cytokeratin family, consisting of 37 genes, using the same methodology for AVS revealed that cytokeratin variance score was similar between HPV16+/p53WT and HPV−/p53HRmut tumors (Supplemental Fig. 5). Based on our findings that AVS is positively correlated with mRNAsi, we speculated that AVS may be a marker for CIC homogeneity such that high AVS is indicative of a tumor populated with homogenous CICs at high frequency. This notion is further supported by our data that the CIC pool was severely contracted as a consequence of targeting the dominant ALDH isoform in two high AVS HNSCC cell lines; one in the HPV16+ setting and the other in the HPV− setting. ALDH isoform specific inhibitors are actively being developed for oncologic indications, however, biomarkers are needed to select patients likely to respond to these targeted molecules. A potential clinical application for AVS is as a niche molecular biomarker to match high AVS HNSCC patients to specific ALDH isoform inhibitors.

## Materials and Methods

### Cell lines

SCC25 and CAL27 cell lines were purchased from ATCC (Manass, VA). UMSCC74A and UMSCC47 cell lines were obtained from Thomas Carey, University of Michigan. UPCI:SCC090 and UD-SCC2 cell lines were provided by Susanne Gollin, University of Pittsburgh and Henning Bier, Heinrich-Heine University, Dusseldorf, Germany. CAL27, UD-SCC2, UMSCC47, UMSCC74A, and UPCI:SCC090 were grown in Dulbecco’s modified Eagle’s medium containing 10% fetal bovine serum, 100 µg/ml streptomycin, and 100 U/ml penicillin. SCC25 cells were grown in a 1:1 mixture of Ham’s F-12 and DMEM supplemented with 10% FBS, 0.4 µg/mL hydrocortisone, 100 µg/ml streptomycin, and 100 U/ml penicillin. Cell lines were maintained in a humidified atmosphere of 5% CO_2_ at 37 °C. Cell lines were authenticated using STR profile analysis and not tested for mycoplasma contamination.

### Quantitative real-time PCR

Cells were extracted for total RNA using the TRIzol reagent (ThermoFisher Scientific, Waltham, MA) or TaqMan PreAmp Cells-to-CT kit (ThermoFisher Scientific). mRNA expression of ALDH isoforms was determined using the Applied Biosystems 7900HT Fast Real-Time PCR System with validated, pre-designed TaqMan primers (ThermoFisher Scientific). The expression of ALDH isoforms were normalized to glyceraldehyde 3-phosphate dehydrogenase (GADPH) using the ∆∆Ct method.

### ALDH variance score

Clinical data from the TCGA HNSCC dataset were downloaded from the Genomics Data Commons data portal/TCGA Research Network: http://cancergenome.hih.gov/. HPV status for the TCGA HNSCC cohort were determined by PCR from the biospecimen center Nationwide Children’s Hospital, and downloaded from the auxiliary file of the clinical TCGA HNSCC dataset from the GDC data portal. All HPV16+ and HPV− cases from the TCGA HNSCC cohort were identified and analyzed. Mutational profiles of individual patients were obtained from the updated Pan-Cancer dataset. p53 missense mutations were classified as high risk using the evolutionary action score of p53 (EAp53) threshold of >75^34^. Pre-processed legacy RNA-seq data were downloaded from NCI Genomic Data Commons using *TCGAbiolinks* package in R and gene level GC-content normalization was performed^35,36^. RNA-Seq by Expectation Maximization (RSEM) of individual ALDH isoforms was converted to percent of total ALDH RSEM by dividing the RSEM for each ALDH isoform by the sum of RSEM from all ALDH isoforms. For each sample, the deviation of each ALDH isoform from the mean percentage was calculated and squared to avoid negative values. ALDH variance score (AVS) was then calculated as an average of the squared deviations using the formula:

$$AVS=\mathop{\sum }\limits_{i=1}^{n}{({x}_{i}-mean(x))}^{2}/n$$

### Stemness index

The PanCancer Atlas published a set of stemness indices based on mRNA expression (mRNAsi) by machine-learning scoring the oncogenic dedifferentiation of TCGA tumor samples^19^. Briefly, the entire RNA expression gene set was evaluated and each gene was weighted based on stemness features. The stemness of each sample was scored accordingly and subsequently mapped to the [0, 1] range.

### Inducible shRNA-ALDH1A3 expression system

Three targeting sequences to knockdown ALDH1A3 were designed, synthesized, and cloned into the inducible tetracycline-on pLV-RNAi (BioSettia, San Diego, CA) vector. Sequence 1: 5′ AAAAGGTCAAGTTCACCAAGATATTGGATCCAATATCTTGGTGAACTTGACC 3′; Sequence 2: 5′ AAAAGCAGAGAACTAGGTGAATATTGGATCCAATATTCACCTAGTTCTCTGC 3′ Sequence 3: 5′AAAAGCAGGTCTACTCTGAGTTTGTTTGGATCCAAACAAACTCAGAGTAGACCTGC 3′. Cells were transduced with pLV-RNAi/shRNA-ALDH1A3 and polyclonal cell populations were collected. Preliminary studies demonstrated that sequence 1 resulted in the most efficient knockdown of ALDH1A3. All experiments in this study were performed using sequence 1 and cells were stimulated with doxycycline at 1 µg/ml.

### Immunoblot

Whole cell lysates were mixed with Laemmli loading buffer, boiled, separated by SDS–PAGE, and transferred to a nitrocellulose membrane. Subsequently, immunoblot analyses were performed using antibodies specific to ALDH1A3 (ab129815; abcam) or GADPH (AB2302; MilliporeSigma) and processed using the Pierce Fast Western Blot Kit (ThermoFisher Scientific).

### ALDEFLUOR assay

Cells were suspended in ALDEFLUOR assay (Stem Cell Technologies, British Columbia, Canada) buffer containing ALDH substrate (bidipy-aminoacetaldehyde, 1 mM per 1 × 10^6^ cells) and incubated for 45 mins at 37 °C. For each experiment, an aliquot of cells was exposed to diethylaminobenzaldehyde (50 mM), an ALDH inhibitor, to serve as the negative control. The sorting gate was established using the negative control as the baseline. Cell suspensions were centrifuged at 300 g for 5 mins at 4 °C and re-suspended in 0.5 mL ALDEFLUOR assay buffer for analysis. Fluorescence-activated cell sorting analyses were performed using BD FACS Calibur (BD Lifesciences, Franklin Lakes, NJ) at The Ohio State University Comprehensive Cancer Center Analytical Cytometry Core.

### Tumorsphere formation assay

Cells were collected and seeded in a serum-free defined medium consisting of keratinocyte serum-free medium supplemented with epidermal growth factor, basic fibroblast growth factor, insulin and hydrocortisone in low-attachment plates (Corning Incorporated, Corning, NY). Tumorsphere formation efficiency was calculated by dividing the number of tumorspheres (≥50 µm in diameter) formed in 7 d by the initial number of cells seeded. Tumorsphere diameter was measured using the NIS-Elements software (Nikon Instruments, Melville, NY).

### *In vivo* tumor incidence

Inducible shRNA-ALDH1A3 UMSCC47 cells were suspended in 50:50 DMEM:Matrigel and implanted subcutaneously into the flank of 6–8 week old, female NOD/SCID mice. Subsequently, for each cell dilution, mice were randomly assigned to two treatment arms; control diet (n = 5) or doxycycline (200 mg/kg)-containing diet (n = 5) *ad libitum*. Tumor incidence was monitored for 49 days following tumor cell implantation. Cancer initiating cell frequency was calculated using the L-Calc program (STEMCELL Technologies Inc., Vancouver, Canada). Sample size estimate was not performed for this experiment. All animals were included in our analysis. Investigative team was not blinded to the group allocation during the experiment and when assessing the endpoint of tumor incidence. Animal experiments were conducted in compliance with ethical regulations and under an approved protocol from The Ohio State University.

### Statistical analyses

All analyses were performed in R. Fisher’s exact, Spearman, Student’s t, or Wilcoxon tests were used when appropriate to evaluate the association between categorical variables. Five-year OS and DSS plots were generated by the Kaplan-Meier method and log-rank test was used to compare the plots. Cox proportional hazards model was used for univariate analysis for individual ALDH isoforms as a continuous variable. All tests were two-tailed and p-values < 0.05 were considered significant.

## Supplementary information

Supplementary Information.
