# Supplementary material for: p53 functional states are associated with distinct aldehyde dehydrogenase transcriptomic signatures
Source: Sci Rep. 2020 Jan 23;10:1097. doi: 10.1038/s41598-020-57758-5 (PMC6978341; doi:10.1038/s41598-020-57758-5)
Supplement: Supplementary file 1 — Supplementary Information. [file 41598_2020_57758_MOESM1_ESM.pdf]

Supplemental Information

p53 functional states are associated with distinct aldehyde dehydrogenase transcriptomic signatures

Shanying Gui, Xiujie Xie, Wendi Q O’Neill, Kate Chatfield-Reed, Jun-Ge Yu,

Theodoros N Teknos, and Quintin Pan

Supplemental Figure 1

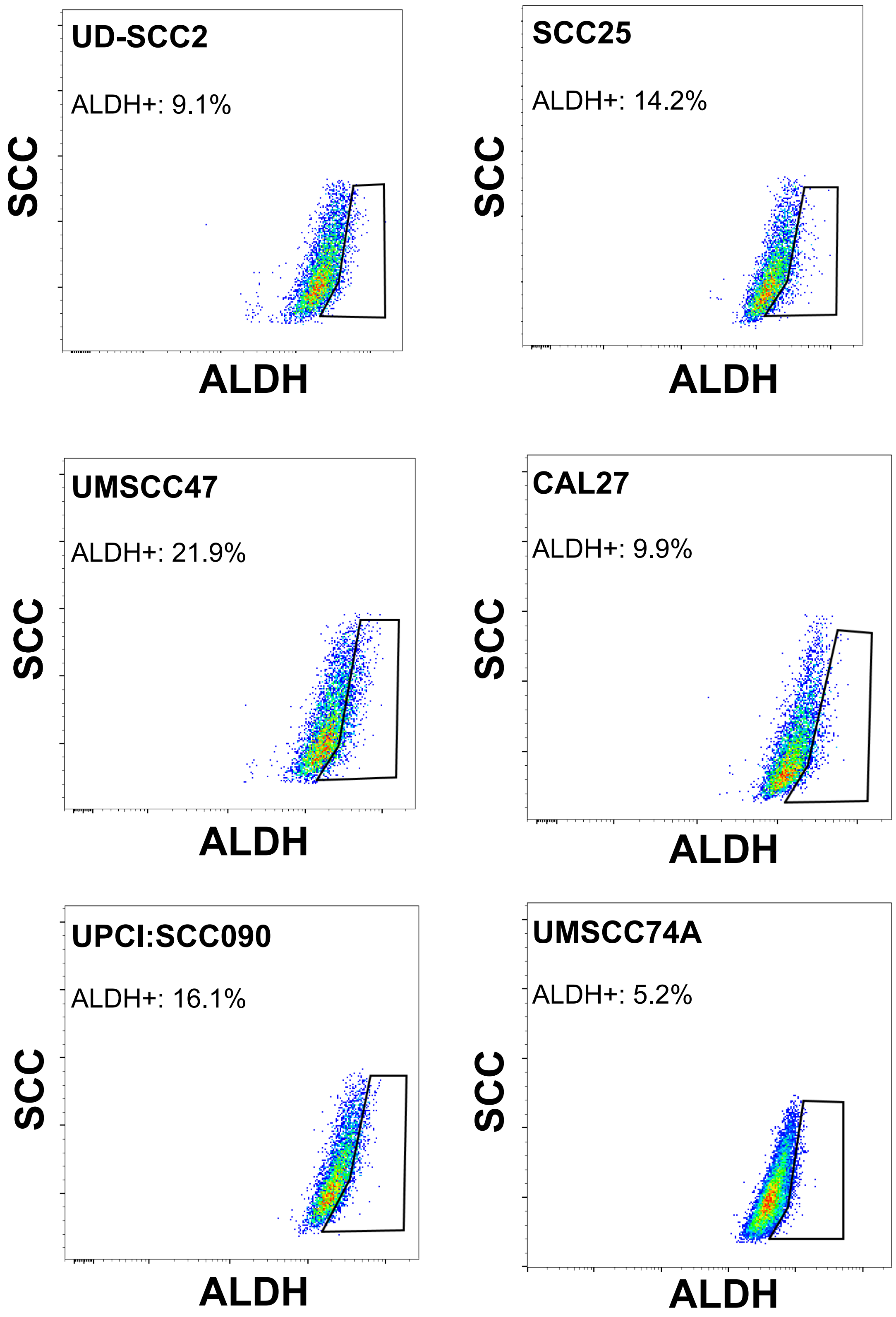

Supplemental Figure 1. ALDEFLUOR assay for HPV16+ and HPV- HNSCC cell lines. Representative FACS plot for each cell line is presented.

Supplemental Figure 2

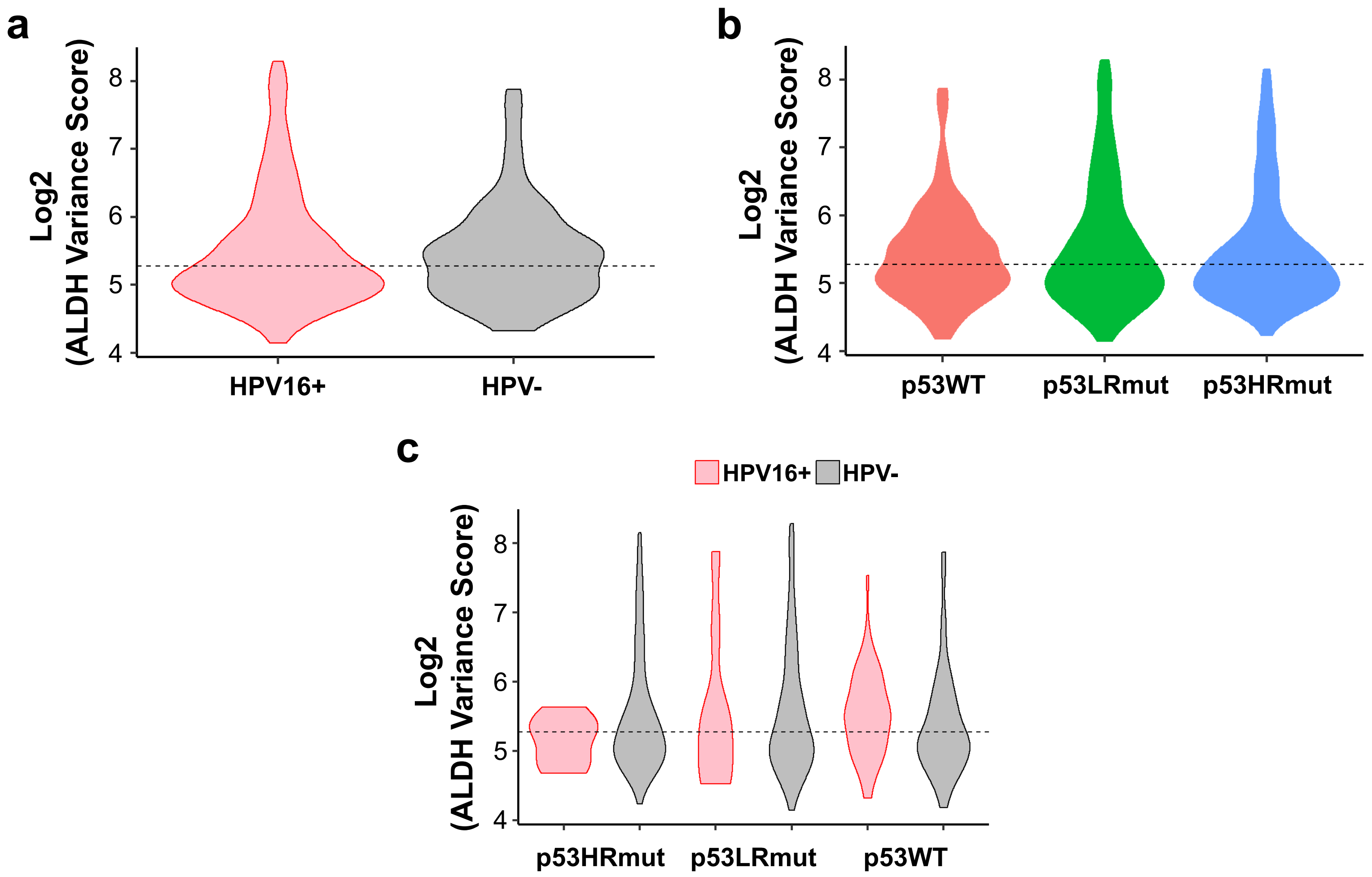

Supplemental Figure 2. Violin plots for AVS distribution in the HPV16+ and HPV- HNSCC TCGA cohort. (a) Cohort stratified based on HPV status. (b) Cohort stratified based on p53 states. (c) Cohort stratified based on HPV status and p53 states.

Supplemental Figure 3

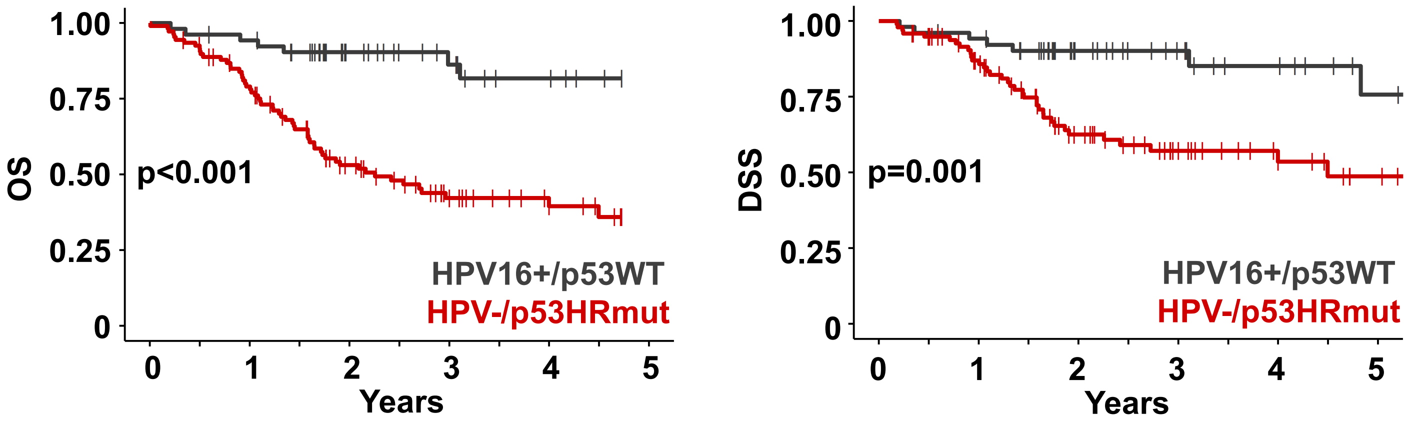

Supplemental Figure 3. Five-year Kaplan-Meier plots for overall and disease-specific survival in HPV16+/p53WT and HPV-/p53HRmut HNSCC patients. Log-rank test was used to compare the Kaplan-Meier plots.

Supplemental Figure 4

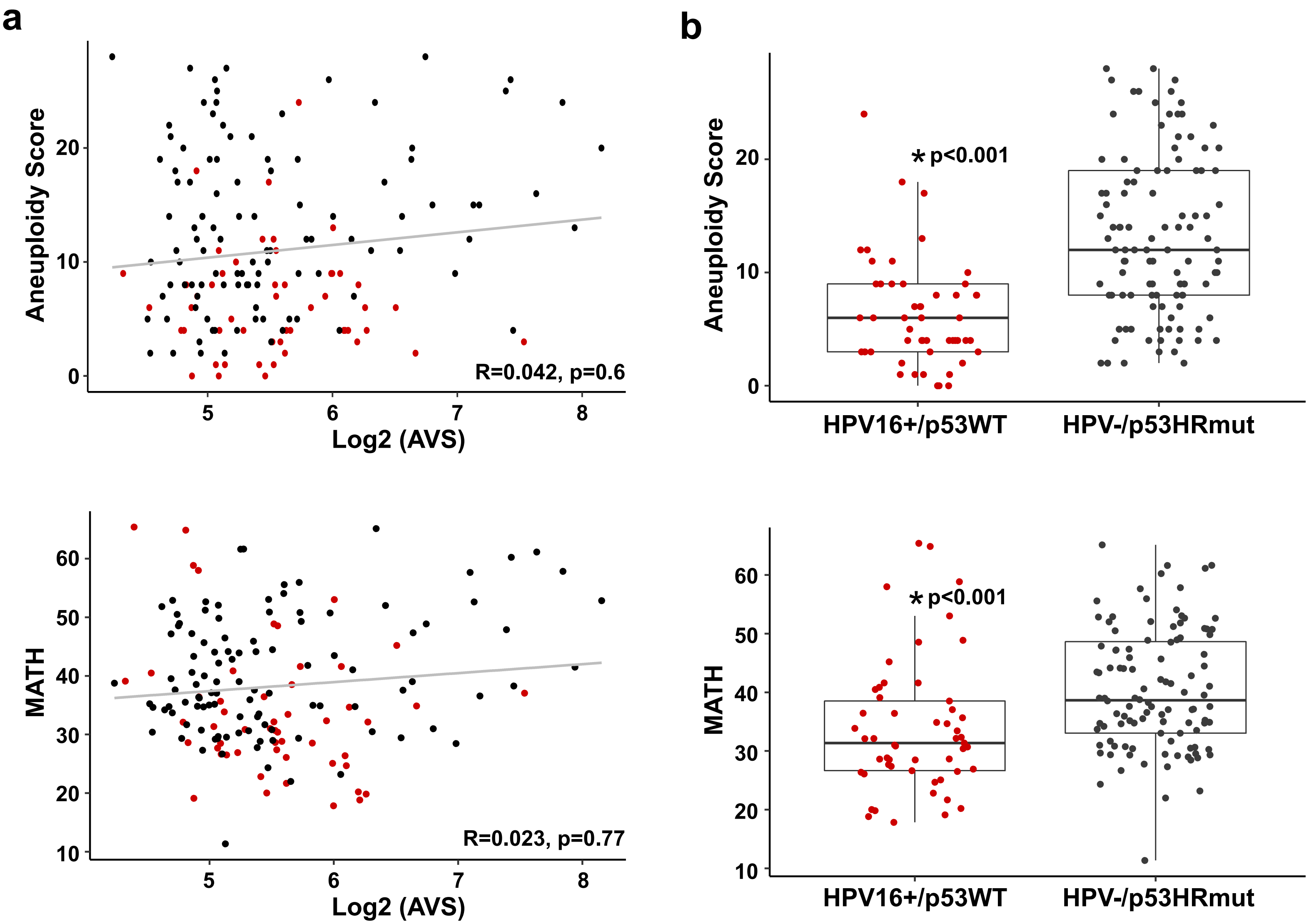

Supplemental Figure 4. Aneuploidy score and MATH. (a) Spearman test was used to determine the association between AVS and aneuploidy acore, and AVS and MATH in the combined HPV16+/p53WT (red dots) and HPV-/p53HRmut (black dots) cohort. (b) Aneuploidy score and MATH are lower in HPV16+/p53WT than in HPV-/p53HRmut HNSCC tumors. Wilcoxon test.

Supplemental Figure 5

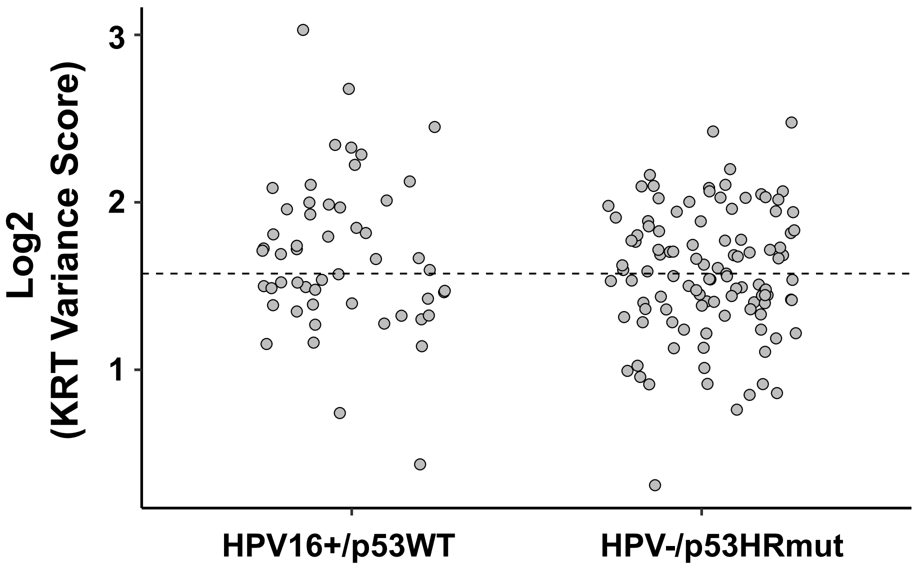

	KRT Variance Score	
	low	high
HPV16+/p53WT	25	28
HPV-/p53HRmut	55	53

p=0.738

Supplemental Figure 5. Cytokeratin (KRT) variance score was calculated using the same methodology as AVS. Dashed line represents the median KRT variance score. Median KRT variance score for the combined cohort was used to stratify into two groups, high and low, and analyzed using Fisher's exact test.

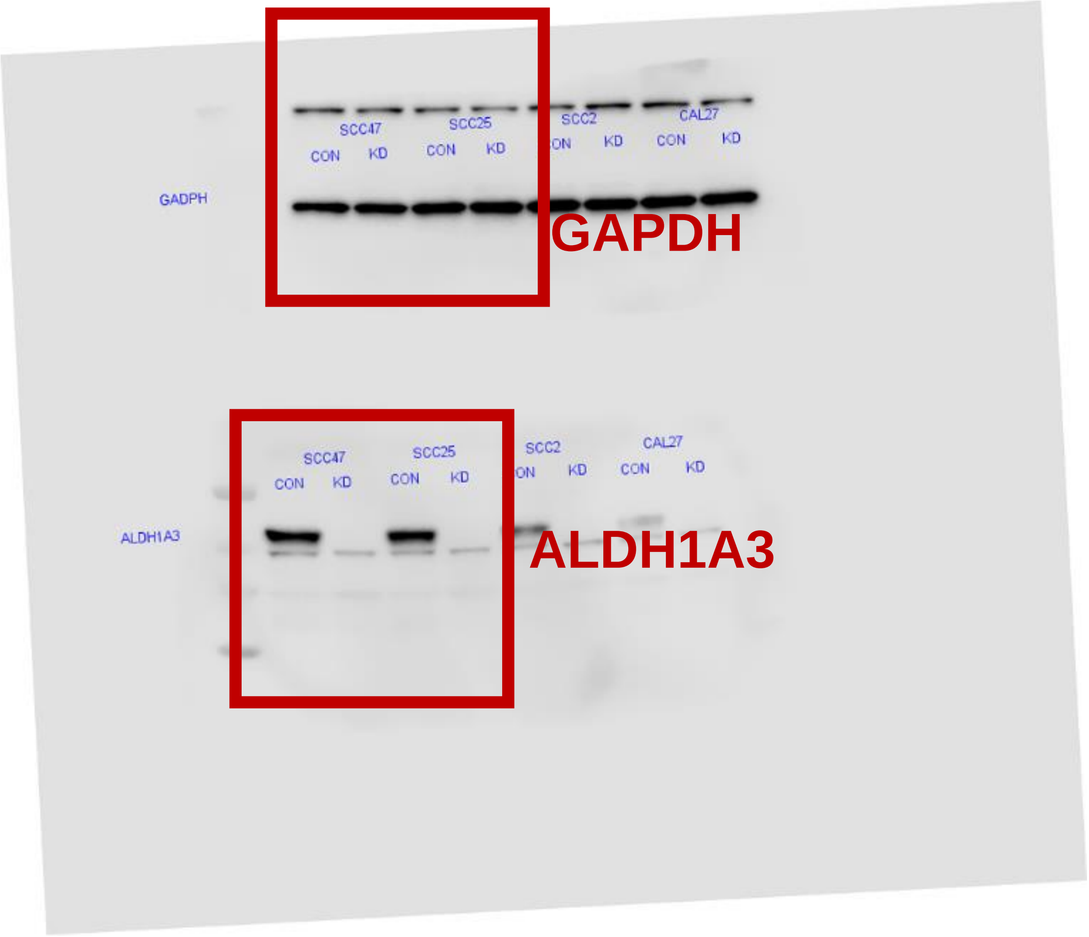
SCC47
CON
KD
SCC25
CON
KD
SCC2
ON
KD
CAL27
CON
KD
GADPH
GAPDH
SCC47
CON
KD
SCC25
CON
KD
SCC2
ON
KD
CAL27
CON
KD
ALDH1A3
ALDH1A3
